# The timing of initiation of pharmacotherapy for women with gestational diabetes mellitus

**DOI:** 10.1186/s12884-020-03449-y

**Published:** 2020-12-11

**Authors:** Rachel K. Harrison, Meredith Cruz, Ashley Wong, Caroline Davitt, Anna Palatnik

**Affiliations:** 1 Department of Obstetrics and Gynecology, Division of Maternal Fetal Medicine, Medical College of Wisconsin, 9200 W. Wisconsin Ave, Milwaukee, WI 53226 USA; 2 Medical College of Wisconsin, 8701 W. Watertown Plank Rd, Milwaukee, WI 53226 USA; 3 Center for Advancing Population Science, Medical College of Wisconsin, Milwaukee, WI 53226 USA

**Keywords:** Gestational diabetes mellitus, Pharmacotherapy, Glycemic threshold, Insulin, Oral hypoglycemic agent

## Abstract

**Background:**

The decision to initiate pharmacotherapy is integral in the care for pregnant women with gestational diabetes mellitus (GDM). We sought to compare pregnancy outcomes between two threshold percentages of elevated glucose values prior to initiation of pharmacotherapy for GDM. We hypothesized that a lower threshold at pharmacotherapy initiation will be associated with lower rates of adverse perinatal outcomes.

**Methods:**

This was a retrospective cohort study of women with GDM delivering in a single tertiary care center. Pregnancy outcomes were compared using bivariable and multivariable analyses between women who started pharmacotherapy (insulin or oral hypoglycemic agent) after a failed trial of dietary modifications at two different ranges of elevated capillary blood glucose (CBG) values: Group 1 when 20–39% CBG values were above goal; Group 2 when ≥40% CBG values were above goal. The primary outcome was a composite GDM-associated neonatal adverse outcome that included: macrosomia, large for gestational age (LGA), shoulder dystocia, hypoglycemia, hyperbilirubinemia requiring phototherapy, respiratory distress syndrome, stillbirth, and neonatal demise. Secondary outcomes included cesarean delivery, preterm birth (< 37 weeks), neonatal intensive care unit (NICU) admission, and small for gestational age (SGA).

**Results:**

A total of 417 women were included in the study. In univariable analysis, the composite neonatal outcome was statistically significantly higher in Group 2 compared to Group 1 (47.9% vs. 31.4%, *p* = 0.001). In addition, rates of preterm birth (15.7% vs 7.4%, *p* = 0.011), NICU admission (11.7% vs 4.0%, *p* = 0.006), and LGA (21.2% vs 9.1% *p* = 0.001) were higher in Group 2. In contrast, higher rates of SGA were noted in Group 1 (8.0% vs. 2.9%, *p* = 0.019). There was no difference in cesarean section rates. These findings persisted in multivariable analysis after adjusting for confounding factors (composite neonatal outcome aOR = 0.50, 95%CI [0.31–0.78]).

**Conclusions:**

Initiation of pharmacotherapy for GDM when 20–39% of CBG values are above goal, compared to ≥40%, was associated with decreased rates of adverse neonatal outcomes attributable to GDM. This was accompanied by higher rates of SGA among women receiving pharmacotherapy at the lower threshold. Additional studies are required to identify the optimal threshold of abnormal CBG values to initiate pharmacotherapy for GDM.

## Background

Gestational diabetes mellitus (GDM) is characterized by abnormal glucose tolerance and is a product of heightened insulin resistance in pregnancy [1, 2]. High-quality evidence has long demonstrated the association of GDM and resulting maternal hyperglycemia with adverse perinatal outcomes [1–8]. Monitoring and treating GDM reduces these adverse outcomes [1–9]. Notably, the extent of treatment needed is based upon the woman’s glycemic response to diet and exercise alone, with nearly 90% of women failing an initial trial of prescribed diet and exercise [2]. However, the definition of what constitutes an unsuccessful attempt at diet and exercise has not yet been established. No randomized controlled trials — or, in fact, any prospective or retrospective studies — have evaluated the optimal glycemic threshold for initiation of pharmacotherapy, in addition to diet and exercise. It is likely that each provider caring for women with GDM decides individually what proportion of elevated capillary blood glucose (CBG) values merits initiation of pharmacotherapy and how rigorously these CBG values should be controlled [2].

The need to solve this clinical question is further elucidated after review of society recommendations, including those of the American College of Obstetricians and Gynecologists (ACOG) and the American Diabetes Association (ADA) [1, 9]. In effect, there is no clear consensus on how to manage the initiation and adjustment of pharmacotherapy for GDM. ACOG states that “...treatment is recommended when target glucose levels cannot be *consistently* achieved through nutrition therapy and exercise” [1]. ACOG further states that there is “no conclusive evidence for a specific threshold value at which medical therapy should be started” [1, 10]. The ADA is similarly vague regarding recommendations for initiating and titrating pharmacotherapy for GDM including statements such as: “there are no adequately powered randomized trials comparing different fasting and post-meal glycemic targets in diabetes in pregnancy” [9].

In light of this identified gap of knowledge and lack of consensus, we designed a retrospective cohort study to compare two different thresholds for pharmacotherapy initiation in women with GDM. We hypothesized that a lower threshold for pharmacotherapy initiation will be associated with improved perinatal outcomes and will not be associated with higher rates of complications.

## Methods

This was a retrospective chart review of women with GDM that were started on pharmacotherapy during pregnancy between 2011 and 2019 at a single academic center. Institutional review board approval was obtained and maternal and neonatal chart review was performed to extract all data pertinent to this study. Women were included in the study if they were pregnant with a viable singleton gestation, at least 18 years of age, and were diagnosed with GDM and started on pharmacotherapy (A2GDM) with insulin or an oral hypoglycemic agent for blood glucose control in pregnancy. Women were excluded if they had A1GDM (not on medications), pre-gestational diabetes, multifetal gestation, fetal anomalies, or missing pregnancy or delivery information. Diagnosis of GDM in our system is assigned after an elevated glucose level 1 h after a 50-g glucose load with a cutoff of ≥140 mg/dL (7.8 mmol/L), followed by two abnormal results after a 100-g glucose load on a 3 h test with cutoffs of ≥95 mg/dL (5.3 mmol/L), ≥180 mg/dL (10.0 mmol/L), ≥155 mg/dL (8.6 mmol/L), and ≥ 140 mg/dL (7.8 mmol/L) at fasting and 1, 2, and 3 h after the 100-g glucose load, respectively. Alternatively, women with a 1-h glucose level of 200 mg/dL (11.1 mmol/L) after a 50-g glucose load are assumed to have GDM as well. Lastly, women started on pharmacotherapy with less than 20% abnormal CBG values were excluded, assuming they were affected by a severe phenotype of GDM or had a high likelihood of pre-gestational diabetes to justify such early pharmacotherapy initiation.

In our hospital system, women who are diagnosed with GDM are initially counseled on diet and exercise in an attempt to control GDM without pharmacotherapy. Women record home CBG values in a log at fasting and at 1 or 2 h after each meal for 1 week per provider or patient preference, for a total of four CBG checks per day. Normal cutoff values were > 95 mg/dL (5.3 mmol/L) fasting and either > 140 mg/dL (7.8 mmol/L) or > 120 mg/dL (6.7 mmol/L) at 1 and 2 h after meals, respectively. The provider then reviews the glucose log and determines whether pharmacotherapy initiation is appropriate at that time. The woman continues weekly collection of CBG values throughout pregnancy, and the provider assesses the need to add pharmacotherapy on a weekly basis. For the purpose of our analysis, the threshold of abnormal CBG values the week preceding the prescription of pharmacotherapy was reviewed and collected. Medications prescribed included Insulin, Glyburide, or Metformin and were started by Maternal-Fetal Medicine specialists, General Obstetrician Gynecologists (OB/GYN), or Endocrinologists who manage GDM.

Maternal and neonatal outcomes were compared between two groups based on the number of abnormal CBG values recorded at the time of pharmacotherapy initiation. Group 1 included women who had 20–39% abnormal CBG values at the time of pharmacotherapy initiation and Group 2 included women who had at least 40% abnormal CBG values at the time of pharmacotherapy initiation. The choice of cutoff of 40% was based on the median and standard deviation of a subset of patients examined at the start of data collection and because it was similar to those used in prior studies [5, 6]. The glucose log data was also collected on a subset of patients.

The primary outcome was a composite neonatal outcome that included the following: macrosomia, defined as birth weight > 4000 g [11], large for gestational age (LGA), defined as birth weight greater than the 90th percentile at birth [12], shoulder dystocia defined as inability to deliver the anterior shoulder requiring additional maneuvers [13], neonatal hypoglycemia, defined as more than one blood glucose value of < 40 mg/dL (2.2 mmol/L), hyperbilirubinemia requiring phototherapy, respiratory distress syndrome (RDS), stillbirth, and neonatal demise. Secondary maternal outcomes were rates of cesarean delivery, preeclampsia, wound infection, third- or fourth-degree laceration, postpartum hemorrhage, defined as estimated blood loss of ≥500 mL for vaginal delivery and ≥ 1000 mL for cesarean delivery [14], and maternal hypoglycemia, defined as maternal CBG less than 70 mg/dL. Secondary neonatal outcomes were rates of NICU admission, preterm delivery (< 37 weeks gestation) and small for gestational age (SGA) infants, defined as birth weight less than the 10th percentile [12, 15]. All tests were two-tailed and *p* < 0.05 defined statistical significance. Univariable comparisons were conducted with Chi-square, Fisher exact, Student’s T test, or Mann-U Whitney as appropriate. Multivariable logistic regression was used to estimate the independent association between the CBG threshold for pharmacotherapy initiation and rates of primary and secondary study outcomes. All analyses were performed with Stata version 14.0 (StataCorp College Station, TX).

## Results

**Fig. 1:**
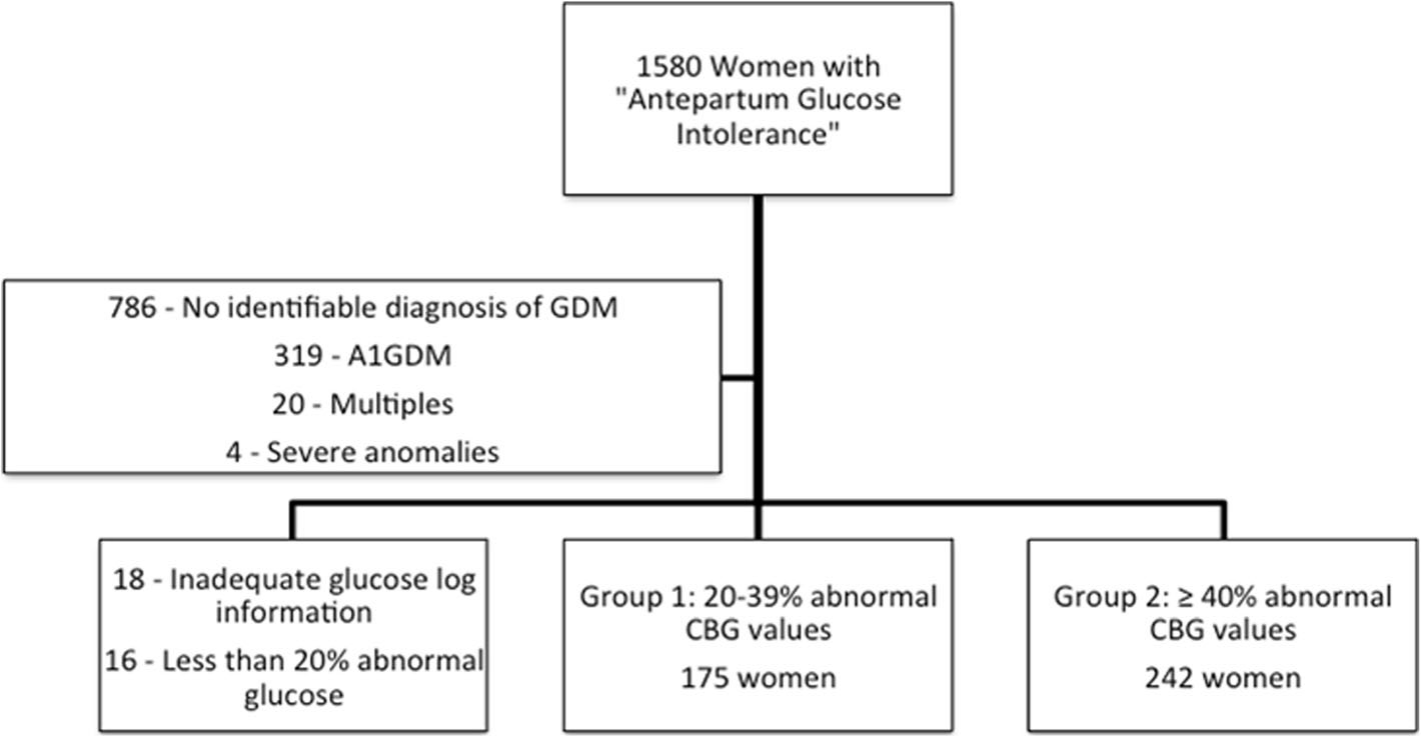
Flow Chart of Subject Selection”. Legend: “GDM: Gestational diabetes mellitus, A1GDM: Diet-controlled gestational diabetes mellitus, CBG: Capillary blood glucose”

**Fig. 2:**
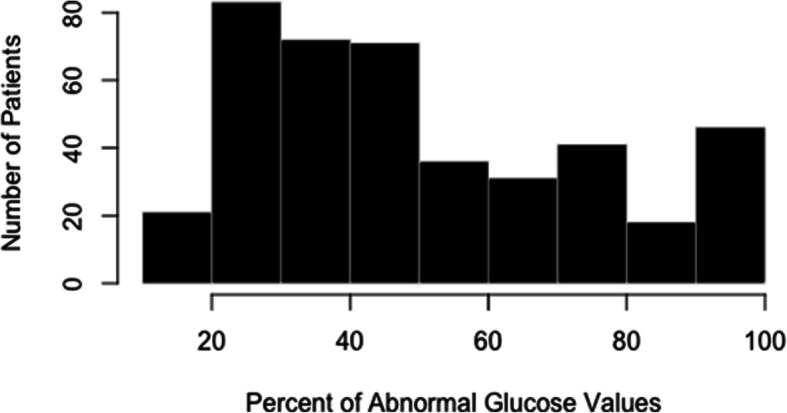
“Percentage of Abnormal Glucose Values at Pharmacotherapy Initiation”

From over 1500 women with a diagnosis code O99.810, “abnormal glucose tolerance in pregnancy”, 417 women met inclusion criteria (Fig. 1). Seven hundred eighty-six women were found to have this code in their electronic medical record, however, these women did not meet inclusion criteria and they were not included as they had no evidence of GDM in the identified pregnancy. Most often on chart review, they were found to have had an abnormal 1-h result with a normal 3-h result, a history of GDM in a prior pregnancy, or incomplete medical record to confirm GDM. The 18 women without an adequate glucose log were women for whom CBG values were never recorded in the medical record and could therefore not be evaluated by our team. The percent of abnormal CBG values prior to initiation of pharmacotherapy ranged from 20 to 100%. The distribution of the percent abnormal CBG values can be seen in Fig. 2, with the majority of patients started on pharmacotherapy at 20–50% abnormal values. One hundred seventy-five women (Group 1) were started on pharmacotherapy at 20–39% abnormal CBG values and 242 women were started on pharmacotherapy at ≥40% abnormal CBG values (Group 2). In Group 1, the mean percent of abnormal values was 29% (standard deviation (SD) = 7) and median of 29% (range 20–39%). In Group 2, the mean percent of abnormal values was 67% (SD = 20) and median of 64% (range 41–100%).

In univariable analysis, women in Group 1 were more likely to be non-Hispanic white (68.0% vs. 47.9%, *p* < 0.001), have a lower BMI at the beginning of pregnancy (32.1 ± 8.3 vs. 35.3 ± 9.3 kg/m^2^, p < 0.001) and be privately insured (67.6% vs. 52.3%, p < 0.001) (Table 1). The managing provider differed between groups as well, with women in Group 1 more likely to be managed by Endocrinologists (32.8% vs. 23.7%, *p* = 0.030) and less likely to be managed by Maternal-Fetal-Medicine specialists (10.9% vs. 18.7%, p = 0.030) (Table 1). Women in both groups were similarly likely to be started on insulin (48.3% vs 46.8%, *p* = 0.773) and the majority of women who were started on oral agents received glyburide (87.6%).

**Table 1:** Baseline and pregnancy characteristics

	Group 1–20-39% abnormal glucose values at pharmacotherapy (***N*** = 175)	Group 2 – ≥40% abnormal glucose values at pharmacotherapy (***N*** = 242)	***p***-value
Age (years)	32.0 ± 4.7	31.5 ± 5.3	0.241
**Early pregnancy BMI (kg/m**^**2**^**)**	**32.1 ± 8.3**	**35.3 ± 9.3**	**< 0.001**
Nulliparity	61 (34.9%)	88 (36.4)	0.751
**Insurance**			**< 0.001**
**Private**	**117 (67.6%)**	**124 (52.3%)**	
**Public**	**32 (18.5%)**	**93 (39.2%)**	
**None**	**24 (13.9%)**	**20 (8.4%)**	
**Maternal race/ethnicity**			**< 0.001**
**White**	**119 (68.0%)**	**116 (47.9%)**	
**Non-Hispanic Black**	**17 (9.7%)**	**62 (25.6%)**	
**Hispanic**	**10 (5.7%)**	**36 (14.9%)**	
**Other**	**29 (16.6%)**	**28 (11.6%)**	
**Marital status**			**0.001**
**Married**	**124 (76.5%)**	**132 (58.9%)**	
**Divorced**	**4 (2.5%)**	**9 (4.0%)**	
**Single**	**34 (21.0%)**	**83 (37.1%)**	
**Oral Glucose Tolerance Test Results**			
**Fasting (mg/dL)**	**92.3 ± 9.9**	**101.0 ± 17.0**	**< 0.001**
**1-h (mg/dL)**	**191.6 ± 25.0**	**201.3 ± 29.4**	**0.005**
**2-h (mg/dL)**	**172.0 ± 27.7**	**185.0 ± 32.7**	**0.001**
**3-h (mg/dL)**	**129.7 ± 36.1**	**142.8 ± 34.1**	**0.011**
Gestational age at diagnosis (weeks)	25.1 ± 6.3	26.2 ± 5.6	0.073
Gestational age at treatment (weeks)	29.0 ± 6.0	29.5 ± 5.3	0.403
**Managing Provider**			**0.030**
**MFM**	**19 (10.9%)**	**45 (18.7%)**	
**General OB/GYN**	**98 (56.3%)**	**139 (57.7%)**	
**Endocrinology**	**57 (32.8%)**	**47 (23.7%)**	
Tobacco use	13 (7.5%)	22 (9.2%)	0.479
Maternal chronic hypertension	10 (5.7%)	10 (4.1%)	0.456
Gestational weight gain (kg)	8.6 ± 6.7	9.6 ± 7.2	0.183
Insulin given as initial treatment	84 (48.3%)	111 (46.8%)	0.773

All data presented as N (%) or mean ± SD, bold indicates statistical significance

*BMI* body mass index, *MFM* maternal-fetal medicine, *OB/GYN* obstetrician gynecologist

Table 2 describes pregnancy outcomes. The composite neonatal outcome was statistically significantly lower in Group 1 than Group 2 (31.4% versus 47.9%, *p* = 0.001). When looking at the individual outcomes, the rates of shoulder dystocia (0.6% vs 5.4%, *p* = 0.007), macrosomia (6.3% vs 12.4%, *p* = 0.039), and LGA (9.1 vs. 21.2%, p = 0.001) were lower in women receiving pharmacotherapy at a lower percent of abnormal CBG values (i.e. Group 1). NICU admission and preterm delivery rates were also lower in Group 1 (4.0% vs. 11.7%, *p* = 0.006 and 7.4% vs. 15.7%, *p* = 0.011, respectively). In contrast, Group 1 had higher rates of SGA infants (8.0% vs. 2.9%, *p* = 0.019). Maternal outcomes did not differ between the two groups, including rates of maternal hypoglycemia (22.9% vs 19.5%, *p* = 0.410) (Table 2).

**Table 2:** Pregnancy Outcomes

	Group 1–20-39% abnormal glucose values at pharmacotherapy (*N* = 175)	Group 2 – ≥40% abnormal glucose values at pharmacotherapy (*N* = 242)	p-value
**Composite neonatal outcome**^**a**^	**55 (31.4%)**	**116 (47.9%)**	**0.001**
5-min Apgar < 7	6 (3.5%)	18 (7.4%)	0.090
**Macrosomia**	**11 (6.3%)**	**30 (12.4%)**	**0.039**
**Large for gestational age**	**16 (9.1%)**	**51 (21.2%)**	**0.001**
**Shoulder dystocia**	**11 (0.6%)**	**13 (5.4%)**	**0.007**
Jaundice requiring phototherapy	10 (5.9%)	22 (9.3%)	0.209
Neonatal hypoglycemia	41 (23.8%)	56 (23.6%)	0.961
Respiratory distress syndrome	3 (1.7%)	12 (5.0%)	0.077
Fetal or neonatal demise	1 (0.01%)	0 (0%)	0.239
Birth weight (grams)	3298 ± 532	3403 ± 601	0.067
**Small for gestational age**	**14 (8.0%)**	**7 (2.9%)**	**0.019**
**NICU Admission**	**7 (4.0%)**	**28 (11.7%)**	**0.006**
**Preterm delivery**	**13 (7.4%)**	**38 (15.7%)**	**0.011**
**Gestational age at delivery (weeks)**	**38.4 ± 1.7**	**38.0 ± 1.8**	**0.009**
Cesarean delivery	72 (41.1%)	103 (42.6%)	0.772
Preeclampsia	17 (9.7%)	32 (13.5%)	0.247
Wound infection	3 (2.1%)	10 (5.0%)	0.160
3rd or 4th degree laceration	3 (1.9%)	4 (1.8%)	0.997
Postpartum hemorrhage	9 (5.4%)	18 (7.7%)	0.364
Maternal hypoglycemia	38 (22.9%)	45 (19.5%)	0.410

All data presented as N (%) or mean ± SD, bold indicates statistical significance

*NICU* neonatal intensive care unit

^a^Included: macrosomia, large for gestational age, shoulder dystocia, jaundice, hypoglycemia, respiratory distress syndrome, stillbirth and neonatal demise

In multivariable analysis, controlling for maternal race and ethnicity, insurance status, early pregnancy BMI, gestational age at the time of GDM diagnosis, provider type, medication utilized, and gestational weight gain, women in Group 1 (20–39% abnormal CBG values) continued to have lower rates of the primary composite neonatal outcome (adjusted odds ratio 0.48, 95% Confidence Interval 0.30–0.77) (Table 3). In addition, rates of LGA, NICU admission, and preterm delivery remained lower in Group 1 (Table 3). Similarly, the finding of higher rates of SGA among women in Group 1 persisted, with adjusted odds ratio of 3.84 (95% Confidence Interval 1.31–11.22). Lastly, glucose log data during the week prior to treatment initiation and  4 weeks after treatment initiation was collected and is depicted in Table 4. The data was available for 301 women, of them, 26 conducted 1-h postprandial testing and 275 women conducted 2-h postprandial testing. Due to the small number of women conducting the 1-h, Table 4 analysis was limited to 275 patients conducting the 2-h. Table 4 shows that blood glucose values prior to initiation of pharmacotherapy in Group 1 were significantly lower, both for fasting and postprandial values, compared to Group 2. Four weeks into pharmacotherapy, comparison of mean fasting and postprandial glucose values shows that the fasting values were significantly lower and within the target range in women in Group 1 compared to Group 2, whose values were slightly above the target range.

**Table 3:** Multivariable analysis for impact of 20–39% vs. ≥40% abnormal CBG values

	OR	95% CI	aOR^**b**^	95% CI
**Composite neonatal outcome**^**a**^	**0.50**	**0.33–0.75**	**0.48**	**0.30–0.77**
5-min Apgar < 7	0.45	0.17–1.16	0.40	0.13–1.25
Macrosomia	0.47	0.23–0.97	0.71	0.30–1.67
**Large for gestational age**	**0.37**	**0.21–0.68**	**0.41**	**0.21–0.81**
Shoulder dystocia	0.10	0.01–0.77	0.20	0.02–1.68
Jaundice requiring phototherapy	0.61	0.28–1.33	0.58	0.23–1.46
Neonatal hypoglycemia	1.01	0.64–1.60	0.89	0.52–1.52
Respiratory distress syndrome	0.33	0.09–1.20	0.37	0.09–1.53
Fetal or neonatal demise	**–**	**–**	**–**	**–**
**NICU Admission**	**0.32**	**0.13–0.74**	**0.38**	**0.15–0.96**
**Small for gestational age**	**2.91**	**1.15–7.36**	**3.84**	**1.31–11.22**
**Preterm delivery**	**0.43**	**0.22–0.84**	**0.38**	**0.18–0.80**
Cesarean delivery	0.94	0.64–1.40	1.04	0.66–1.65
Preeclampsia	0.69	0.37–1.29	0.85	0.42–1.73
Wound infection	0.40	0.11–1.49	0.64	0.15–2.75
3rd or 4th degree laceration	1.00	0.22–4.55	1.40	0.24–8.06
Postpartum hemorrhage	0.68	0.30–1.56	0.84	0.32–2.21
Maternal hypoglycemia	1.19	0.73–1.94	1.12	0.63–1.99

Bold indicates statistical significance

*NICU* neonatal intensive care unit

^a^Included: macrosomia, large for gestational age, shoulder dystocia, jaundice, hypoglycemia, respiratory distress syndrome, stillbirth and neonatal demise

^b^Controlled for maternal race, early pregnancy body mass index, gestational age at diagnosis, insurance, managing provider, initial medication, gestational weight gain

**Table 4:** Capillary blood glucose values before and after pharmacotherapy initiation

	Before pharmacotherapy initiation			After pharmacotherapy initiation		
	Group 1 (*N* = 111)	Group 2 (*N* = 164)	*P*-value	Group 1 (*N* = 111)	Group 2 (*N* = 164)	*P*-value
Fasting CBG (mg/dL, mmol/L) (*N* = 2850)	93.8 ± 12.6	108.7 ± 19.5	<.001	89.6 ± 11.1	96.6 ± 21.5	0.009
	5.2 ± 0.7	6.0 ± 1.1		5.0 ± 0.6	5.4 ± 1.2	
2-h postprandial CBG (mg/dL, mmol/L) (*N* = 6786)	116.8 ± 19.6	131.6 ± 27.9	0.002	115.32 ± 22.7	126.7 ± 34.6	0.021
	6.5 ± 1.1	7.3 ± 1.5		6.4 ± 1.3	7.0 ± 1.9	

## Discussion

In this study we found that pharmacotherapy initiation for women with GDM at the threshold of 20–39% abnormal CBG values, compared to a threshold of ≥40%, was associated with higher likelihood of reaching target range for fasting and postprandial CBG values 4 weeks after initiation of pharmacotherapy. In addition, it was associated with lower odds of a composite neonatal outcome comprised of GDM-related complications including macrosomia, LGA, shoulder dystocia, jaundice requiring phototherapy, hypoglycemia, RDS, stillbirth and neonatal demise. Preterm delivery rates were also reduced in the 20–39% threshold group; however, rates of SGA were increased. Furthermore, we were able to demonstrate that women who were in the lower threshold group had lower fasting and postprandial glucose values that were sustained 4 weeks after treatment initiation (Table 4).

Prior studies, including a systematic review by Poolsup et al. and two landmark trials by Crowther et al. and Landon et al., have established that treatment of women with GDM leads to a reduction in adverse maternal and neonatal outcomes [5, 6, 8]. Nonetheless, the threshold at which pharmacotherapy was added differed between the studies as a 14% cutoff of abnormal CBG values was used in the Crowther et al. study and a 50% cutoff was used in the Landon et al. study [5, 6]. Other studies, including those by Garner et al. and Niromanesh et al. [16, 17], initiated treatment after two abnormal values, whereas many of the remaining studies evaluating GDM management either did not specify at which glycemic threshold pharmacotherapy was started, or assigned women randomly to immediate pharmacotherapy without a trial of diet and exercise [18–21].

It is important to establish safe and effective criterion for pharmacotherapy initiation for GDM as under-treatment may increase GDM-related complications [1–8]; whereas overtreatment may come at increased cost due to overutilization of resources [1, 22–25], increased patient expense [22–24], and adverse effects of the medications themselves, such as maternal hypoglycemia [17, 26–29] and SGA [8, 29]. Based on our results, treatment at a lower threshold of 20–39% abnormal CBG values did not increase the risk of maternal hypoglycemia or other maternal complications, however, the rates of SGA were significantly increased compared to women who received pharmacotherapy at a threshold of 40% abnormal CBG values. Langer et al. identified a similar phenomenon demonstrating higher rates of SGA infants in women with lower mean glucose levels while undergoing treatment for GDM [29]. When evaluating Table 4, we see evidence of this phenomenon as well as fasting and all 2-h postprandial levels were lower in women in Group 1 (the more intensively treated group) when compared to Group 2 after 4 weeks of treatment.

In contrast, poorly controlled maternal diabetes leads to accelerated fetal growth attributable to fetal hyperinsulinemia, a product of maternal hyperglycemia [11, 30, 31]. High insulin levels trigger fetal overgrowth due to its hypertrophic effects [30, 31]. Fetuses experiencing lower levels of insulin may therefore be susceptible to slower rates of and even suboptimal growth [11, 29, 31]. Overall, even though rates of SGA associated with the CBG threshold of 20–39% were higher, the baseline prevalence of SGA in this group of women with GDM was lower than the 10% reported rate in the literature and below rates seen in the general population [15]. This is consistent with the overall effect of GDM on newborn weight [1, 2, 31].

Regarding maternal outcomes, we did not find a significant reduction in the rates of preeclampsia, cesarean delivery, postpartum hemorrhage, third- and fourth-degree lacerations, or wound infection. This is likely related to our sample size and overall higher risk of these complications in all women with GDM, regardless of pharmacotherapy initiation. Additional limitations of our study include lack of information regarding patient compliance with treatment and the exclusion of women who remained A1GDM, i.e. diet controlled. We did not include this cohort, as our goal was to study women that received pharmacotherapy and currently there are no clear guidelines concerning when to add pharmacotherapy to GDM management. Furthermore*,* we do not have income or educational information on the subjects to assess the full impact of socioeconomic status on our findings; however, maternal non-Hispanic White race, specialist access, and insurance status appear to be related to initiation of treatment at lower thresholds. Thus, the impact of these factors deserves further attention in future studies. Lastly, this is a retrospective analysis and therefore no causal influences can be drawn. Strengths of the study include the novelty of the study question and the gap in the literature we have addressed regarding pharmacotherapy initiation for GDM.

## Conclusions

In summary, we identified that pharmacotherapy initiation for women with GDM at a lower threshold of abnormal CBG values of 20–39% is associated with improved neonatal outcomes, with the exception of a higher risk of SGA. Future directions of this research should include a prospective randomized controlled trial comparing feasibility and efficacy of different cutoffs for the ideal percent of abnormal CBG levels prior to pharmacotherapy initiation.

## Data Availability

The datasets used and/or analyzed during the current study are available from the corresponding author on reasonable request.

## Abbreviations

GDM: Gestational diabetes mellitus
CBG: Capillary blood glucose
SGA: Small for gestational age
NICU: Neonatal intensive care unit
ACOG: American College of Obstetricians and Gynecologists
ADA: American Diabetes Association
GDMA1: Gestational diabetes mellitus diet controlled
GDMA2: Gestational diabetes mellitus medication controlled
OB/GYN: Obstetrician Gynecologists
LGA: Large for gestational age
RDS: Respiratory distress syndrome
BMI: Body mass index
